# Evaluating an Emergency Department Discharge Center: A Learning Organization Approach for Efficiency and Future Directions

**DOI:** 10.7759/cureus.73470

**Published:** 2024-11-11

**Authors:** Bibi S Razack, Naya B Mahabir, Lisa O Iyeke, Lindsay Jordan, Helena Willis, Marina Gizzi-Murphy, Frederick Davis, Adam J Berman, Mark Richman, Nancy S Kwon

**Affiliations:** 1 Emergency Medicine, Valley Stream Hospital, Valley Stream, USA; 2 Emergency Medicine, Northwell Health Long Island Jewish Medical Center, New Hyde Park, USA; 3 Internal Medicine, Icahn School of Medicine at Mount Sinai, New York City, USA

**Keywords:** discharge process, emergency department, emergency medicine, evaluation, learning organization, social determinants of health, transitions of care, vulnerable patients

## Abstract

Introduction

Our pilot Emergency Department Discharge Center (EDDC) facilitates post-discharge appointments, and screens for social determinants of health (SDoH) with a long, paper-based tool. No criteria guide which patients to refer to EDDC for appointment-making. Patients screening positive for SDoH are texted or emailed a list of community-based organizations (CBOs) to contact; the screening tool doesn’t assess patients’ interest or ability to contact CBOs. Additionally, our ED’s clinical and operational administrators run a follow-up call program for discharged patients to inquire about their recovery. This program is associated with improved patient satisfaction, a strategic initiative tied to reimbursement. Owing to high volume, only 8.6% (4,877 of 56,591) of discharged patients are called. We describe an application of Learning Organization principles and practices to evaluate EDDC efficiency and identify opportunities to create time for EDDC staff to participate in and expand the follow-up call program.

Methods

A “Learning Organization” follows five principles (systems thinking, personal mastery, mental models, shared vision, and team learning) to facilitate its members’ learning and continuously transform itself. To evaluate EDDC processes (“systems thinking”), the overriding Learning Organization principle we adopted was “integrate learning into the business process.” We established “team learning” by engaging EDDC staff and ED leadership (“leadership commitment”), thereby “promoting ownership at every level.” We shadowed EDDC staff and analyzed data for 3,616 patients receiving appointment assistance, 342 offered SDoH screening, and 4,877 called by phone. We identified the validated SHOUT tool (which predicts discharge failure) and its highly weighted criteria (no home, insurance, or primary care physician). We randomly surveyed 50 patients to determine: 1) what percent met those highly-weighted criteria, with the idea being to guide providers about which patients particularly benefit from EDDC assistance, and 2) what percent had not only SDoH social service needs but also interest and ability to contact CBOs, as this would be their responsibility. Adopting these two changes (SHOUT tool and assessing interest/ability to contact CBOs) might yield more judicious utilization of EDDC personnel, freeing up time to staff the follow-up call program.

Results

EDDC staff spend ~35 minutes/patient. They don’t make appointments but instead liaise with physicians’ offices, which yields fewer ED returns and admissions. Only 6% (3 of 50) of surveyed patients met SHOUT criteria for EDDC assistance. Of 342 patients screened for SDoH, 31% (106) completed the survey, 20% (68) identified a need, and only 4.5% (15) completed it, identified a need, and followed up on their own after receiving CBO names and contact information. Only 50% of call-back patients were contactable: 77% had improved, 21% were unchanged; ~50% had made appointments without EDDC assistance; and 12.5% had clinical questions.

Conclusion

Learning Organization exercises identified the SHOUT tool and revealed the potential for SHOUT criteria and QR-code-accessible two-step SDoH surveys to create significant time for EDDC to staff follow-up program expansion. Thousands more patients would be screened for SDoH, saving 95% of the effort while retaining 100% of the benefit. EDDC staff would serve as a safety net for follow-up calls for patients unable to secure an appointment.

## Introduction

Timely follow-up after discharge from the ED improves patient-centered outcomes such as 30-day admissions [1]. However, after discharge, many patients have difficulty securing and attending appointments due to challenges including lack of insurance, misunderstanding discharge instructions, and overlooking the importance of follow-up care [2]. Many ED patients lack a primary care physician (PCP) or specialist, making it difficult to access timely healthcare [3]. Even when patients have a PCP or specialists, these providers are often unaware their patients visited an ED [4]. Patients with a PCP or specialist may still have difficulty arranging their own following-up on account of lack of appointment unavailability (a situation unlikely to improve based on projections of physician supply and demand) [5], inadequate insurance, poor health literacy, mental health disorders, low self-efficacy, or non-adherence to their treatment plan [6,7,8]. Patients discharged who leave the ED with only a phone number rather than a specific appointment are less likely to attend a discharge appointment within 10 days [9].

In addition, social factors (e.g., access to food, housing, immigration services, transportation, etc.) contribute significantly to health outcomes [10]. Addressing such social determinants of health (SDoH) through interventions targeting a wide range of factors, including education and early childhood, urban planning and community development, housing, income enhancements and supplements, and employment, has been demonstrated to decrease health disparities [11].

In July 2020, Northwell Health Long Island Jewish (LIJ) Medical Center Emergency Department ED administrators instituted a pilot Emergency Department Discharge Center (EDDC) to 1) improve patient follow-up after ED discharge by having care coordinators facilitate patient follow-up appointments prior to discharge, and 2) increase the screening for SDoH and referral to CBO resources.

Detailed information about the EDDC is presented in our previous work [12]. Briefly, the EDDC operates Monday to Friday, from 7 AM to 5 PM, and occasionally Saturdays, and is staffed by care coordinators. There are no criteria/guidelines or restrictions on characteristics of patients (e.g., age, insurance, diagnoses) who can be referred to the EDDC. A survey of 30 Long Island Jewish Medical Center (LIJMC) ED providers found great variation in patients whom they refer to the EDDC, including patients who: 1) need a PCP or specialist, 2) require 1-2-week follow-up, 3) have poor health literacy, 4) don’t speak English, 5) already had difficulty making an appointment, and 6) would be admitted if not for a timely appointment [12].

In our previous work, we performed a univariate/unadjusted analysis using the student’s T-test to determine the association between EDDC use and 72-hour ED returns and 30-day admissions (with statistical significance a priori at <0.05). EDDC patients were slightly less likely to return to the ED within 72 hours (number needed to treat (NNT) = 83)) or be hospitalized within 30 days (NNT = 143) compared with patients not referred to the EDDC. These effects were driven by assisting profoundly-vulnerable patients (elderly, uninsured, no PCP). During the EDDC pilot year, 3,616 patients were managed by the EDDC (6.4% of all 56,591 discharged patients). EDDC staffing has since increased and is on pace to assist 12,000 patients this year (16% of ~75,000 discharges).

EDDC staff also screen patients for SDoH using a lengthy, printed form. Responses indicating a social service need (e.g., housing, transportation) are given to an ED secretary, who manually enters patient identifiers, contact information and particular social service needs data into a commercially available social services portal (formerly NOWPOW®, currently Unite Us®), which then texts or emails the names and contact information of local non-profit CBOs. Patients are responsible for following up themselves. That secretary then enters largely the same information into a separate ED-created, HIPAA-compliant REDCap® database. An internal review of operational data revealed that, during the pilot year (during which there were 56,591 ED discharges), EDDC staff screened 565 patients (1% of discharges) for SDoH. They are on pace to screen ~5,000 patients this year (6.7% of discharges). Given the extensive resources that would be required to “shepherd” patients through the CBO follow-up process, there are no plans for the EDDC pilot program to adopt that function/responsibility, despite patients who are unwilling or unable to contact CBOs themselves being those who would most benefit from CBO assistance.

The ED has a separate, non-EDDC program to create a post-discharge safety net and improve patient satisfaction. For the past 2 years, the ED has operated a follow-up call program staffed by physician and physician assistant administrators. Patients are queried within one week of discharge about the course of their condition, whether they have follow-up appointments, and if they have visit-related questions. This program is associated with higher satisfaction scores, an ED strategic initiative tied to reimbursement [13]. Consequently, ED administrators aim to expand the program to reach all discharged patients. Patients are selected to be called if they were: 1) discharged from the ED, 2) are English-speaking, and 3) are still on the ED billers/coders follow-up board 1-2 days post-discharge. The follow-up board is the means by which billers/coders notify providers their note is missing some element (e.g., physical examination); providers frequently check the follow-up board to resolve those deficiencies and, having done so, remove the patient from the follow-up board. Hence, administrators making call-backs have at their disposal only the random subset of English-speaking patients in whom providers have not yet addressed documentation deficiencies. In addition, patients often do not answer the call, further randomly reducing the number of successfully completed call-backs. For these reasons, plus high volume, only a random, small percent of patients (8.6%, 4,877 of 56,591) were called by a combination of ED physicians (60% (2,920 calls)), nurses (23% (1,125 calls)), or administrative staff (17% (832 calls)).

ED administration aimed to improve EDDC efficiency (defined as decreasing the NNT for 72-hour returns and 30-day admissions and screening more patients for SDoH) while expending fewer resources (i.e., staff time) on appointment-making assistance and SDoH screening. More targeted work by EDDC staff could free up time to expand the follow-up call program by delegating EDDC staff (i.e., non-clinical staff) to perform such calls. EDDC staff could then serve as a “safety net” for patients by assisting those contacted during a follow-up call who have been unable to make post-discharge appointments.

To investigate how we might improve EDDC efficiency, we adopted a “Learning Organization” mindset and methodology. A “Learning Organization” is one that follows MIT Sloan School of Business’ Peter Senge’s five principles, which he delineated in 1990 as systems thinking, personal mastery, mental models, shared vision, and team learning) to “facilitate the learning of its members and continuously transform itself” [14]. In 2008, Harvard Business School professors Garvin, Edmondson, and Gino further described key features of a “Learning Organization:” having a supportive learning environment, concrete learning processes and practices, and leadership that reinforces learning [15]. Below, we describe the process by which we utilized “Learning Organization” principles to evaluate the EDDC for opportunities for improved efficiency.

This article was previously posted to the medRxiv preprint server on August 1, 2024.

## Materials and methods

LIJ is a 583-bed hospital serving a racially and socioeconomically diverse population that includes many uninsured or underinsured patients. The adult ED sees ~100,000 patients and discharges ~75,000 patients annually.

Our learning/discovery team consisted of ED physicians and nursing administrators, research faculty, and EDDC staff. We established “team learning” by engaging EDDC staff and ED administrators (“leadership commitment”), thereby “promoting ownership at every level” [14].

We first examined existing processes. To begin, we shadowed EDDC staff as they received and responded to requests to assist with appointment-making. We discovered there is no standard method for referring a patient to the EDDC. An ED provider can refer nth patient by (i) walking to the EDDC and asking care coordinators to visit the patient in the ED at the bedside; (ii) calling the EDDC and asking a care coordinator to visit the patient in the ED at the bedside; (iii) walking the patient to the EDDC; (iv) indicating via the “Sunrise®” EHR “ED Provider Note” (disposition section) that the “patient requires follow-up;” (v) emailing the EDDC. We observed EDDC staff manage incoming cases from each of these referral mechanisms, reaching out to the patient both in-person and via telephone and contacting appropriate clinics.

**Figure 1:**
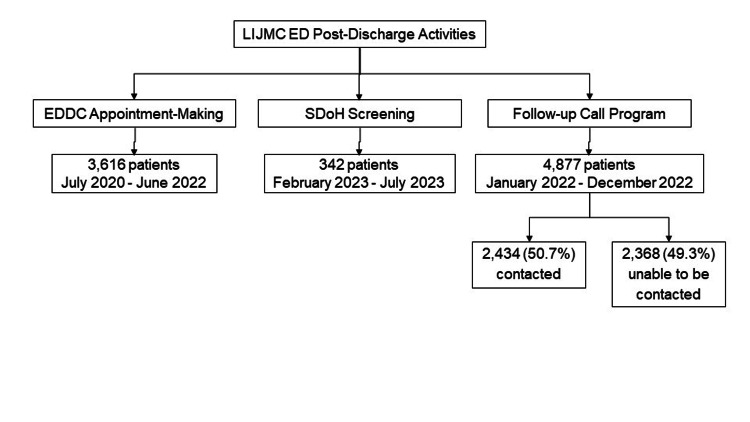
Flow diagram for LIJMC ED post-discharge activities and populations LIJMC: Long Island Jewish Medical Center; ED: Emergency Department; EDDC: Emergency Department Discharge Center; SDoH: social determinants of health

We conducted a retrospective review of EDDC data. Patients were included if they had been referred to the EDDC during the pilot year (July 2020 - June 2021) or subsequent year (July 2021 - June 2022). Appointment-making data is stored in the EDDC’s Microsoft Excel® database (Microsoft Corporation, 2021, Redmond, WA) and was analyzed in Microsoft Excel®. SDoH and follow-up call data were stored in Northwell’s REDCap® database (Vanderbilt University, 2004) and downloaded into Microsoft Excel® for analysis. Data included 3,616 patients receiving appointment assistance between July 2020 and June 2022 and 342 offered SDoH screening between February and July 2023. Finally, we analyzed data from 4,877 patients called as part of the follow-up program during its pilot year (January 2022 - December 2022) to assess the effectiveness of this program in identifying patients who would benefit from expedited medical care, such as those who decompensated since ED discharge, had difficulty obtaining appointments, or had clinical questions regarding their ED visit. Of those, 2,434 (50.7%) were successfully contacted and 2,368 (49.3%) were unable to be contacted (Figure 1).

We then turned attention to identifying means to decompress the EDDC’s current workload while continuing to provide high-quality services to patients who would truly benefit from EDDC intervention. We suspected many patients referred to the EDDC for appointment-making assistance likely do not benefit from EDDC services because they have the means to organize their own post-discharge appointments (e.g., they already have a PCP or a specialist, or at least have insurance (which provides them a network of providers with whom to follow up)). Assisting patients who won’t benefit from EDDC assistance may account for the relatively high NNTs for 72-hour returns and 30-day readmissions (i.e., increase the number of patients needed to find one who benefits compared with patients not exposed to EDDC assistance).

Consequently, we performed a literature search seeking a validated screening tool or survey to identify risk factors for discharge failure (defined as a short-term return to the ED). We discovered such a screening survey: the SHOUT tool [16]. The SHOUT tool considers risk factors for discharge failure; risk factors are weighted by points (e.g., male = 2 points, homeless = 7 points). We reviewed the SHOUT tool to determine if a pared-down version could be used to identify patients most likely to benefit from EDDC assistance. Through our review, we learned the highly-weighted SHOUT tool items were: 1) being homeless (7 points), 2) lacking health insurance (10.5 points), and 3) lacking a PCP (21 points)). These characteristics are similar to those of the profoundly vulnerable patients (uninsured, no PCP) we discovered in our analysis of the EDDC’s pilot year benefited most from EDDC appointment-making assistance in decreasing 72-hour returns and 30-day admissions.

We also expected many patients screening positive for SDoH screening would not follow up with CBOs, as our current process puts the obligation on patients to reach out to the CBOs. While, ideally, EDDC staff would reach out to the CBOs on behalf of the patients, doing so would place a significant time burden on EDDC staff, limiting their ability to facilitate appointment-making and screen additional patients for SDoH.

We subsequently randomly surveyed 50 patients to determine: 1) what percent met highly-weighted SHOUT criteria, with the idea being to guide providers about which patients particularly benefit from EDDC assistance, and 2) what percent had not only SDoH social service needs but also interest and ability to contact CBOs, as this would be their responsibility. As the intent of this random survey was to inform the EDDC pilot program redesign, we chose a modest sample size. We aimed to learn whether EDDC staff could use a shorter, two-stage SDoH screening tool, with the first stage being two brief questions assessing whether: 1) the patient has any social-related needs, and 2) if they would be interested in and able to contact CBOs. Patients self-identifying as having a need and being able/interested to contact CBOs would then view the second part of the SDoH survey: a list of specific social needs (e.g., housing, transportation, etc.). The intent of this series of questions would be to screen out patients who would not benefit from EDDC attention given the current policy and procedure require patients themselves to contact CBOs. Therefore, patients (even those with social-related needs) who self-identify as uninterested or unable to contact CBOs would not benefit from the lengthier screening tool regarding their specific needs. While such patients are a population who might especially benefit from CBO assistance, if they are unable or unwilling to contact CBOs, it would not be an efficient use of EDDC staff time to identify their needs with greater specificity.

This study was deemed exempt by the Northwell Health Human Subject Protection Program - Institutional Review Board as a quality improvement project.

## Results

Key observations

Learning Organization exercises revealed the EDDC has four hired staff, of which three are working for eight hours any given day. EDDC staff spent an average of 34.5 min/patient. This was calculated as follows: given holidays and vacation time, we estimated a total of ~288,000 working minutes per year. EDDC staff spend about ⅔ of their time facilitating appointments; the remaining ⅓ of their time is spent on administrative and SDoH activities. EDDC staff noted that, of the 5,563 patients referred to them, 35% either do not want or do not need appointment-making assistance or are unreachable by phone. With approximately 124,800 minutes per year spent on the 3,616 patients whose appointments were facilitated by EDDC staff; we estimate that EDDC staff spent an average of 34.5 min/patient. This time comprises interviewing the patient, reaching out to clinics, and entering data. To the surprise of many people on the research/discovery team, we learned that EDDC staff don’t actually make appointments. Rather, they act as liaisons, calling and/or emailing physicians’ offices to facilitate appointment scheduling on behalf of the patient while verifying the clinic accepts the patient’s insurance. Depending on the procedures at each clinic (e.g., Cardiology Clinic at LIJMC, Pulmonology Clinic at a satellite location), the EDDC may either provide the clinic with the patient’s identifiers and contact information for the clinic to reach out to the patient or may provide the patient with the clinic’s location and phone number for the patient to reach out to the clinic. During the pilot year, it was not part of the EDDC staff’s responsibility to track patients through to their post-discharge appointment. Therefore, of the 3,616 patients who received assistance with making post-discharge appointments, it is not clear how many patients (and, consequently, what percent) fulfilled the intended outcome of the program: to have a completed appointment.

**Figure 2:**
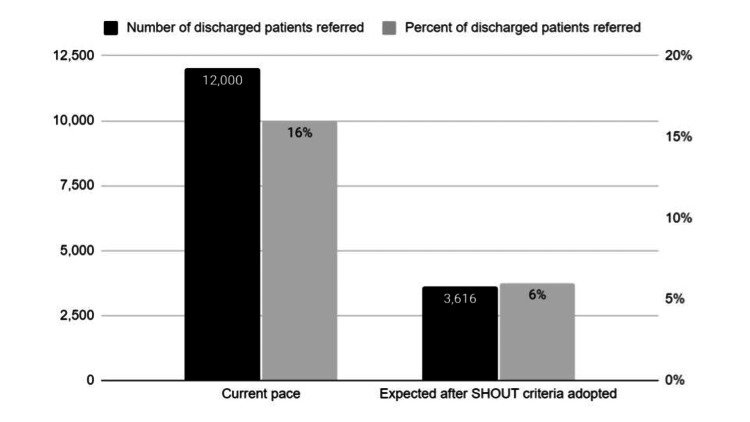
Percent and number of current vs. expected future EDDC referrals EDDC: Emergency Department Discharge Center

The random survey revealed only 6% of patients (3 of 50) meet SHOUT tool criteria for EDDC appointment assistance services, whereas, given the current pace of referrals this year and (lack of) criteria, 16% (12,000 of ~75,000) will be referred to the EDDC) (Figure 2).

**Figure 3:**
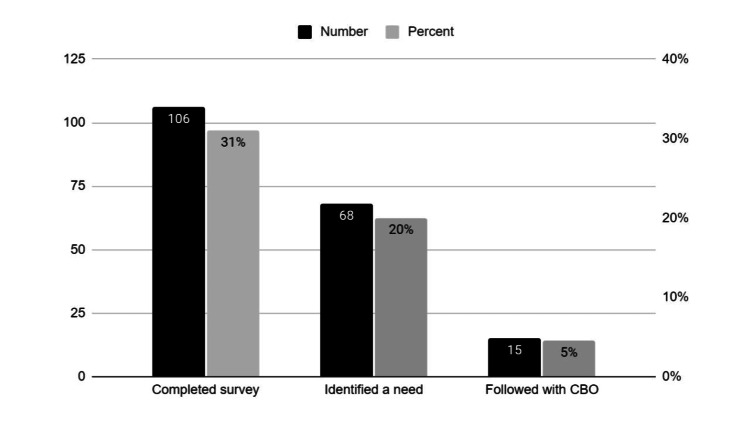
Social determinants of health screening results of 342 patients CBO: community-based organization

Regarding SDoH screening, of all 342 patients offered the screening survey, only 31% (106) completed it; 20% (68) identified a need (65% of those who completed it); and only 4.5% (15) fulfilled the intent of the SDoH screening program by completing the survey, identifying a need, and following-up on their own after receiving texted or emailed CBO names and contact information. The ED secretary manually enters data for all patients who identify a social service need into the ED-created REDCap® database, despite low rates of patients availing themselves of CBOs to which they are referred (that is, only one-quarter of those referred to a CBO follow-through with contacting that CBO). We discovered the ED-created REDCap® database is largely superfluous, as it is never queried and its data is not used for planning or evaluation; for example, the ED has no plans to hire a social worker to address specific needs entered into REDCap® (Figure 3).

**Figure 4:**
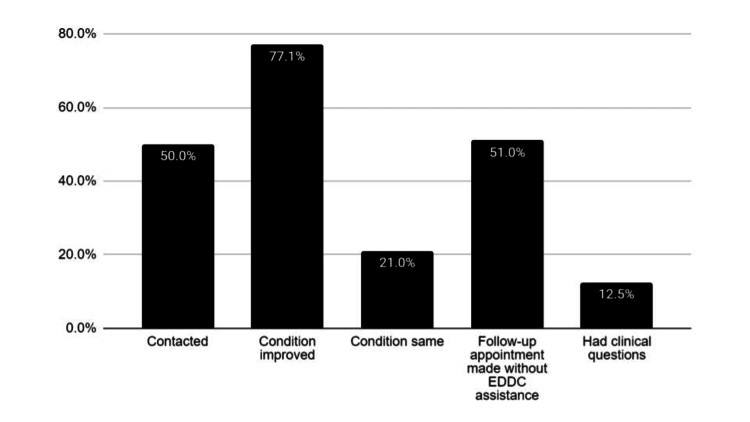
Results from 2,434 patients contacted on follow-up calls EDDC: Emergency Department Discharge Center

Data from 2,434 follow-up call patients who were successfully contacted found 98% (2,385) of patients stated the problem for which they attended the ED had either improved or was the same (77.1% (1,877) = better, 21.0% (511) = same); 51% (1,233) had gotten a 1-week follow-up appointment without EDDC assistance; only 12.5% (304) had clinical questions (Figure 4).

## Discussion

The “Learning Organization” model guided our approach to evaluating our ED’s post-discharge services.

Lessons learned

EDDC staff help facilitate appointments by contacting clinics, verifying insurance, and addressing barriers to follow-up. While the patient might not leave with a time and date for the follow-up appointment, they will leave either with a clinic’s contact information or be in the physician’s office’s queue to call back to help arrange.

The EDDC is associated with a small, but statistically significant, difference in 72-hour returns (NNT = 83) and 30-day admissions (NNT = 143). The absence of criteria for referral to the EDDC likely contributes to these high NNTs, as many patients who won’t benefit from the EDDC’s appointment-facilitating service (because they have a PCP or specialist or can make their own appointments) are referred to the EDDC, inflating the NNT.

Of the 342 patients offered SDoH screening, only 4.5% (15) accomplished the program’s intent by following up on their own after receiving texted or emailed CBO names and contact information. With such low rates, in order to assist a substantial number of patients, screening will have to become more widely distributed and electronic, rather than paper-based. Only 22.5% of patients (15 of 68) screening positive for an SDoH followed through with texts or emails connecting them to CBOs. Characteristics of discharged ED patients at risk for SDoH overlap significantly with characteristics of patients who revisit the ED within 72 hours or are admitted within 30 days: uninsured or having public insurance, mental health disorders, difficulty with transportation and housing, etc. [6,8,17]. Patients unable or unwilling to contact CBOs might likewise have difficulty making or attending outpatient appointments, and, therefore, either utilize the ED for routine care or have worsening chronic conditions that lead to repeat ED visits. Providers might selectively have not referred to the EDDC patients without housing or reliable use of phone service, as the EDDC and clinics would have been unable to contact them to arrange an appointment. Consistent with that observation, none of the 30 providers surveyed identified homelessness or unreliable phone service as a criterion for utilizing the EDDC [12].

We determined that EDDC staff are a flexible resource whose efforts can be more effectively targeted and who can be deployed for a wider variety of purposes than they are currently used (Table 1). Utilizing highly weighted SHOUT tool items to guide referral to the EDDC for appointment-making, and a QR-enabled SDoH screening survey, may improve EDDC staff’s return on effort invested by decreasing the NNT (i.e., the number needed to be “touched” by the EDDC) to prevent one 72-hour return or 30-day admission. This can aid in decreasing ED visits, which is key to reducing the prevalence and harm of ED “boarding” and overcrowding, which contribute to morbidity, mortality, and patient, provider, and staff dissatisfaction [18,19].

**Table 1:** EDDC roles EDDC: Emergency Department Discharge Center; SDoH: social determinants of health

EDDC Role	Existing vs. New Role
Assistance making appointments	Existing
SDoH screening and connection to resources	Existing
Track patients through their post-discharge appointments to determine the efficacy of the appointment-making program not only in facilitating the appointment but through to the program’s goal: having a completed appointment	New
Track patients through their post-discharge appointments to determine the efficacy of the appointment-making program not only in facilitating the appointment but through to the program’s goal: having a completed appointment	New

Future directions

The Learning Organization evaluation process found that adopting SHOUT criteria for screening to the EDDC for appointment assistance would improve efficiency, reducing referrals by ~⅔ (from 16% to 6%, equivalent to 7,500 fewer low-yield referrals). This would free 2,500 hours for EDDC to staff the call-back program, serving as a first-line screen, with the minority of patients with clinical questions referred to a provider.

Because only a few percent of patients complete the SDoH survey, identify a social need, and are willing/able to contact a CBO, the best way to find patients who might benefit from SDoH screening would be to screen many more than only the currently screened <10% of discharged patients. SDoH screening could be expanded by using an electronic screening questionnaire (accessible via QR code in the ED) by which the patient can self-identify as meeting three criteria indicating they would benefit from EDDC assistance (willingness to complete the survey, having a social need, and willingness/ability to contact a CBO) before EDDC staff are utilized. Patients unable to access the QR code or complete the electronic screening questionnaire on their own could be assisted by EDDC staff.

With this in mind, the group decided to make operational changes to improve EDDC efficiency. Specifically, we will create one brief, QR code-accessible electronic tool incorporating SHOUT criteria and SDoH screening to be presented to patients via posted flyers and distribution by clerks. Patients with any highly-weighted SHOUT risk (homelessness, no PCP, uninsured) will be eligible for EDDC assistance. The SDoH portion will have two steps, utilizing branching logic; patients will first be asked if they have any social service-related needs (without inquiring which ones) and are willing and able to contact CBOs on their own. Those unable will not be asked further questions. Those indicating both a need for social services and a willingness/ability to contact CBOs on their own will then be presented with a checkbox list of specific needs (e.g., food, housing, transportation). Only patients who complete the survey, identify a social need, and are willing and able to follow through on their own would occupy EDDC resources. Patients unwilling to complete the QR code survey, who do not identify any social needs, or who are unwilling or unable to follow through on their own will never come to the EDDC staff’s attention. This will save 95% of the effort while retaining 100% of the benefit. Such changes will have the additional benefit of eliminating redundant data entry by having patients enter SDoH data directly into REDCap®, rather than write the information on a form, and then have a secretary enter that information into REDCap®.

With the time saved through these activities, EDDC staff will participate in the follow-up call program, for which they will be trained. In this role, they can serve as an additional safety net for vulnerable patients: follow-up call patients occasionally note difficulty obtaining appointments, an issue with which the EDDC staff are ideally suited to assist.

Our EDDC evaluation process prompted our ED to establish a “Learning Organization” with the features identified by Garvin et al. (2008): a supportive learning environment, concrete learning processes and practices, and leadership that reinforces learning. Other EDs, clinics, and hospitals stand to gain much from our ED’s “Learning Organization” approach to evaluating operations in the realm of post-discharge care (Table 2). We will draw on these elements to assess the effectiveness of the proposed changes. Going forward, ours (and other EDs) can apply “Learning Organization” processes to other ED processes, as well, such as triage and patient flow [20], overcrowding, and boarding [18,19], which lend themselves to broad stakeholder involvement, deep dives, data analysis, and innovative thinking [21].

**Table 2:** Lessons for other organizations

Lessons for other organizations
Overtly state “leadership commitment” to the process
Engage all levels of staff through regular meetings to “integrate learning into the business process” and create “team learning”
Don’t focus on the performance of any particular individual. Instead, adopt a “systems thinking” approach
Listen to front-line thinkers, thereby “promoting ownership at every level”
Use open-ended questions, asked in a spirit of inquiry, not authority or punishment
Dig deep: utilize techniques such as “5 Whys” [11] to uncover underlying issues.
Collect data, but only what will be useful for planning or evaluation.
Review data and stories
Change operations based on data

Limitations

Because this pilot program began during the first (peak) year of the COVID pandemic (when people were avoiding ED [22] and outpatient [23] visits), overall ED volume (and referrals to the EDDC and from the EDDC to PCPs and specialists) may have been lower than they otherwise would have been. In addition, the analysis that described the success of the EDDC in reducing 72-hour returns and 30-day admissions was univariate, not multivariate, and did not adjust for confounding variables that might have influenced the observed differences in 72-hour revisits and 30-day admissions seen, such variables as age, insurance, and absence of a PCP. The original EDDC study followed patients for only 30 days and focused on rates of return ED visits and inpatient admissions, whereas the benefits of attending post-ED appointments might extend beyond 30 days and include non-healthcare benefits such as decreased work absenteeism, slower disease progression, improvement in function/ability to perform activities of daily living (ADLs), etc. Our survey’s small sample size (50 patients) raises the possibility that some findings were more likely to have occurred by chance. For example, only 6% of surveyed patients (3 of 50) met SHOUT tool criteria for referral to EDDC. A larger sample size may have revealed a higher percentage of patients, as it may be by chance alone on the small sample size that only this small percent met the proposed SHOUT criteria for EDDC referral. We did not consider that some patients might not have a cell phone. However, this is uncommon, as 97% of the adult population has a cell phone [24], although the elderly frequently have barriers accessing or using such technology, such as from lack of knowledge, poor technology-related communication skills, concern the technology is too complicated, physical disabilities, or impaired cognition [25]. Similarly, many patients might have a cell phone, but be unable to use it, on account of such conditions as altered mental status or stroke. However, such patients are unlikely to be discharged, and, therefore, would not be candidates for the EDDC appointment-making, SDoH, or follow-up program. For admitted patients, LIJMC has a robust inpatient social work and care manager program that includes SDoH screening and discharge appointment-making. Finally, our program characteristics are unique to LIJMC. Our description of the program, the evaluation process, and our findings might not be applicable to other EDs with Discharge Center-like activities such as appointment-making, SDoH screening, or follow-up calls, as their programs may function differently. For example, other such programs may have criteria defining appropriate patients for Discharge Center services, may utilize electronic SDoH screening, or outsource such activities to a centrally organized health system appointment or social services center.

## Conclusions

Adopting a Learning Organization lens to “facilitate members’ learning and continuously transform” led to insights regarding current and possible future LIJMC EDDC operations to improve EDDC efficiency and overall, ED patient satisfaction via expanding the follow-up call program. Notably, the use of SHOUT criteria to guide the referral of patients to the EDDC might result in a higher return on effort invested in appointment-making assistance. Additionally, an electronic, two-stage SDoH screening process could direct EDDC staff efforts to focus on patients who both need social services and are willing/able to avail themselves of them. Upon implementing these changes, we will assess the impact on the number of patients served by the EDDC for SDoH screening, follow-up calls, and appointment-making, identifying if such changes result in lower NNTs for 72-hour revisits and 30-day admissions. A positive unintended side effect of employing EDDC staff in the follow-up call program is that should a patient inform EDDC staff of difficulty obtaining an appointment, the staff can immediately assist with this issue.
